# Case report: Extravascular catheter migration in a child: A rare complication of the totally implantable venous access device

**DOI:** 10.1097/MD.0000000000032710

**Published:** 2023-02-22

**Authors:** Xuning Lu, Yueyi Ren, Hao Wan, Qiteng Xu, Shuhua Duan

**Affiliations:** a Heart Center, Dalian Municipal Women and Children’s Medical Center (Group), Liaoning, China; b Heart Center, Qingdao Women and Children’s Hospital, Qingdao University, Qingdao, Shandong, China.

**Keywords:** child, complication, extravascular migration, pleural effusion, totally implantable venous access device

## Abstract

**Background:**

Totally implantable venous access devices (TIVADs) are widely used to gain intermittent central venous access, such as in patients who need long-term chemotherapy, total parenteral nutrition, and long-term antibiotic treatment. At present, there are many complications associated with the use of these devices. Complete extravascular migration of TIVADs via the internal jugular vein is a very rare and potentially serious condition, especially in children.

**Case presentation:**

A 1-year-old girl needed palliative chemotherapy because of hepatoblastoma complicated by inferior vena cava thrombosis. A TIVAD was implanted through the right internal jugular vein with a routine heparin flushing tube. On the second day after the operation, a pale bloody liquid was drawn out from the device and the chest X-ray was checked to confirm that the position of the catheter was normal. On the third day after the operation, however, the patient’s right respiratory sound was weakened on physical examination and auscultation. Fluoroscopy showed that the tip of the catheter was located in the right thoracic cavity, and there was a large amount of effusion in the right thoracic cavity. The pleural effusion was removed, the TIVAD was replaced again, and the child was discharged 2 days later.

**Conclusions:**

Following TIVAD implantation, if abnormalities are found, in addition to chest X-ray, saline flush and echocardiography should be performed to determine the position of the catheter and rule out extravascular migration of the catheter to avoid irreparable consequences.

## 1. Introduction

Total implantable venous access devices (TIVADs) play an important role in the treatment of tumors in children. It can provide access for long-term infusion of chemotherapeutic drugs, gastrointestinal nutrition support and antibiotics. The device has many advantages and greatly improves the comfort and safety of patients.^[1]^ However, many complications of the device have been reported in the literature.^[2]^ Early complications may include pain, pneumothorax, hemothorax, catheter dislocation, etc, while late complications can include catheter rupture, intravascular displacement, infection, port leakage, thrombosis, etc^[3,4]^; however, there are few reports on extravascular migration of catheters and pleural effusion in children. Here, we report a case of massive pleural effusion in a child with extravascular migration through the right internal jugular vein (IJV) of a catheter implanted using the Seldinger technique.

## 2. Case presentation

**Figure 1.**
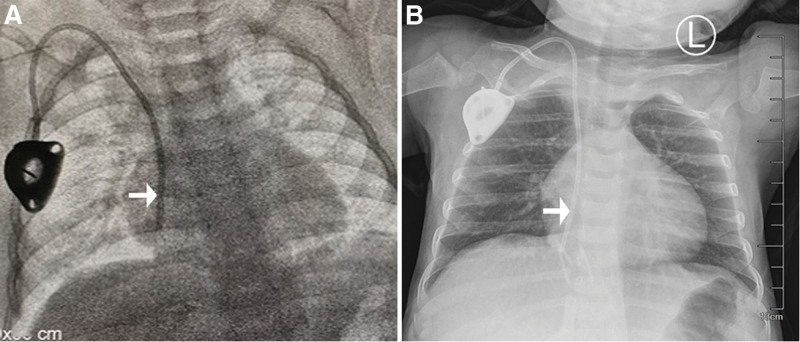
Intraoperative and postoperative images of the first catheterization. (A) Fluoroscopy shows the tip of the catheter (white arrow) in the right atrium. (B) Chest roentgenogram shows the tip of the catheter (white arrow) in the right atrium.

**Figure 2.**
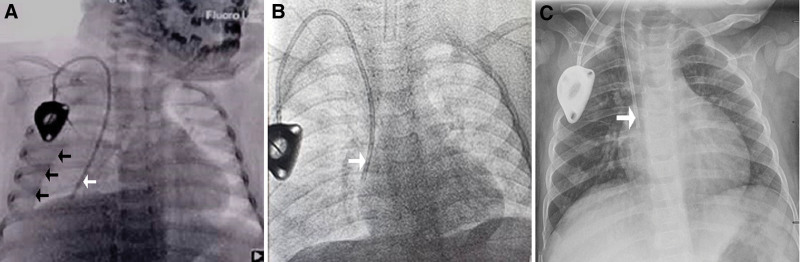
Intraoperative and postoperative images of the second catheterization. (A) Fluoroscopy shows fluid accumulation (black arrows) in the right pleural space with passive atelectasis. The tip of the catheter (white arrow) was in the right pleural space. (B) Fluoroscopy shows the tip of the catheter (white arrow) in the right atrium. (C) Chest roentgenogram shows the tip of the catheter (white arrow) in the right atrium.

A 1-year-old girl was diagnosed with hepatoblastoma with inferior vena cava thrombosis, and a TIVAD was placed to facilitate chemotherapy. The catheter was implanted through the right IJV with the Seldinger technique under ultrasound visualization. During the implantation, the depth of the venipuncture needle was deeper than that of the conventional needle insertion, and the position of the catheter tip was confirmed under fluoroscopy (Fig. 1A). Blood was drawn out smoothly from the device, the whole operation lasted approximately 20 minutes, and no complications occurred during the operation. After the operation, antibiotics were infused through the device, blood was routinely drawn back before use, and the device was normal. On the second day after the operation, a pale bloody liquid was drawn out from the device and the chest X-ray was checked to confirm that the position of the catheter was normal (Fig. 1B). Because of the patient’s young age, the child was crying inconsolably after the operation. On the third day after the operation, however, the patient’s right respiratory sound was weakened on physical examination and auscultation. Fluoroscopy showed that the tip of the catheter was located in the right thoracic cavity, and there was a large amount of effusion in the right thoracic cavity (Fig. 2A). The pleural effusion was removed, and the TIVAD was replaced through the ipsilateral IJV. Under fluoroscopy, the catheter position was reconfirmed (Fig. 2B). The child was discharged from the hospital 2 days after the operation, and the chest X-ray was checked again before discharge to confirm that the position of the catheter was normal (Fig. 2C).

## 3. Discussion

The placement of TIVAD can be divided into 2 methods according to how the device is implanted in the central vein.^[5]^ In the surgical incision approach, which generally utilizes the cephalic vein or external jugular vein, the catheter is implanted into the vein under direct vision, and the tip of the catheter is placed at the junction of the right atrium and superior vena cava.^[6]^ The percutaneous implantation method uses the Seldinger technique, and generally utilizes the subclavian vein (SCV) or IJV,^[7]^ with the reservoir typically placed under the clavicle on the same side. Opinions on which method is better are not uniform. There is no significant difference in overall mortality, catheter displacement, TIVAD-related thrombosis or postoperative bleeding between the 2 methods, and both methods are safe and easy to perform.^[8]^ Klaiber et al^[8]^ noted that compared with percutaneous SCV catheterization, cephalic vein incision implantation technology reduces the risk of pneumothorax and hemothorax. However, Orci et al^[9]^ indicated that the incidence of pneumothorax in patients with surgical incision and closed catheterization was similar, and the success rate of closed catheterization was higher.

It is safe and feasible to choose the SVC approach or IJV approach in closed catheterization.^[10]^ However, the SVC approach is prone to “pinching-off syndrome,”^[11]^ which was first described by Aitkena and Minton based on a 1984 case of “pinching-off syndrome” of catheter rupture and embolism on chest X-ray.^[12]^ Pinching-off syndrome is mainly attributed to the anatomical position of the SVC approach. The catheter must enter the SVC through the gap between the clavicle and the first rib. The gap is wide during catheterization, but it narrows if the patient is in a special position or if the upper limb moves violently, which mechanically squeezes the catheter, resulting in catheter clamping or breaking. To avoid catheter rupture in SCV catheterization, many scholars suggest that TIVADs should be inserted through the right IJV.^[13]^ In addition, through the right IJV approach, the TIVAD can be implanted under ultrasound visualization, and the success rate is higher than that of unguided SCV catheterization. Moreover, catheterization under ultrasound visualization can achieve accurate vein puncture and catheter location, avoid repeated vein puncture, reduce vein injury, and detect catheter angulation or injury in time.^[14]^

To the best of our knowledge, this is the first report of complete extravascular migration of the catheter and pleural effusion through the right IJV approach in a child. Huang et al^[15]^ previously reported a case of complete extravascular migration of the catheter and pleural effusion through the right SCV approach in a child. We analyzed the possible causes of this complication as follows. First, the position where the catheter entered the right IJV was too low, resulting in the short length of the intravascular catheter, which was easily completely detached from the blood vessel. Second, children are prone to crying, and breath holding during crying can cause a sudden increase in intrathoracic pressure. Studies have shown that increased intrathoracic pressure (such as coughing, sneezing, exercise) can cause catheter migration.^[16,17]^ Third, the penetration of the puncture needle was too deep, which damaged the pleural cavity. After the puncture needle entered the normal depth, there was still no blood reflux in the drawing back, thus the needle continued to be inserted forward until the blood reflux was visible in the drawing back. We determined that, in this process, the puncture needle entered the venous lumen from the pleural cavity.

In addition, when the catheter is dysfunctional, a chest X-ray may not be able to accurately confirm the location of the catheter. In this case, while a pale bloody liquid was drawn out from the device and the position of the catheter had been shifted, but the chest X-ray was checked to confirm that the position of the catheter was normal. Extravascular migration of the catheter was confirmed only after a large amount of pleural effusion occurred on the third day after operation. We believe that when the function of the catheter is abnormal, to confirm the position of the catheter, in addition to chest X-ray, saline flush and echocardiography^[18]^ should be performed to accurately confirm the location of the catheter and avoid misdiagnosis.

Complete extravascular migration of the TIVAD to the pleural cavity can cause serious complications, especially during the injection of cytotoxic drugs.^[19]^ Fortunately, the patient was treated with a routine antibiotic that did not cause serious complications. Therefore, when using TIVAD, if abnormalities are found, in addition to chest X-ray, saline flush and echocardiography should be performed to determine the position of the catheter, especially when the language expression ability of the child is poor. In addition, in closed catheterization, attention should be given to the depth of the puncture needle. If there is no blood reflux after the puncture needle reaches the conventional depth, a repuncture should be performed.

## 4. Conclusions

Complete extravascular migration of TIVAD to the pleural cavity in children is a rare and serious complication. When applying TIVAD, on the 1 hand, we should pay attention to the depth of needle insertion when puncturing the vein; on the other hand, before using the catheter, the function of the catheter must be carefully evaluated, and the catheter should be pumped back routinely. In addition to chest X-ray, saline flush and echocardiography should be performed to determine the position of the catheter.

## Acknowledgments

We extend sincere thanks to the patient and his mother for their cooperation to present this case report.

## Author contributions

**Conceptualization:** Xuning Lu.

**Data curation:** Xuning Lu, Yueyi Ren, Hao Wan, Qiteng Xu, Shuhua Duan.

**Formal analysis:** Xuning Lu, Yueyi Ren, Shuhua Duan.

**Investigation:** Xuning Lu, Hao Wan, Shuhua Duan.

**Methodology:** Xuning Lu, Shuhua Duan.

**Resources:** Shuhua Duan.

**Writing – original draft:** Xuning Lu, Shuhua Duan.

**Writing – review & editing:** Xuning Lu, Shuhua Duan.
